# 1-(4-Cyano­phenyl­diazen-2-ium-1-yl)-2-naphtholate

**DOI:** 10.1107/S1600536809028438

**Published:** 2009-07-29

**Authors:** Yan-Hong Yu, Kun Qian

**Affiliations:** a Jiangxi Key Laboratory of Organic Chemistry, Jiangxi Science and Technology, Normal University, Nanchang 330013, People’s Republic of China; b Academic Administration of Jiangxi University of Traditional Chinese, Medicine, NanChang 330047, People’s Republic of China

## Abstract

In the mol­ecule of the zwitterionic title compound, C_17_H_11_N_3_O, the naphthalene ring system is planar [maximum deviation = 0.029 (3) Å] and is oriented at a dihedral angle of 3.55 (3)° with respect to the benzene ring. An intra­molecular N—H⋯O hydrogen bond results in the formation of a planar six-membered ring. In the crystal structure, inter­molecular C—H⋯O inter­actions link the mol­ecules into centrosymmetric dimers.

## Related literature

For general background to azo compounds and their use in dyes, pigments and advanced materials, see: Lee *et al.* (2004 ▶); Oueslati *et al.* (2004 ▶). For a related structure, see: Rădulescu *et al.* (2006 ▶). For bond-length data, see: Allen *et al.* (1987 ▶).
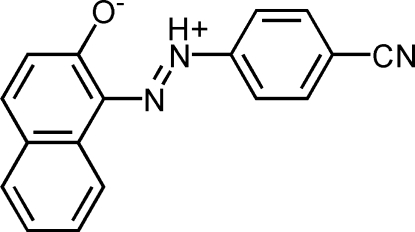


## Experimental

### 

#### Crystal data

C_17_H_11_N_3_O
*M* *_r_* = 273.29
Monoclinic, 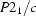

*a* = 5.2673 (11) Å
*b* = 9.910 (2) Å
*c* = 25.239 (6) Å
β = 96.13 (3)°
*V* = 1309.9 (5) Å^3^
*Z* = 4
Mo *K*α radiation
μ = 0.09 mm^−1^
*T* = 294 K
0.35 × 0.10 × 0.10 mm

#### Data collection

Rigaku SCXmini diffractometer
Absorption correction: multi-scan (*CrystalClear*; Rigaku, 2005 ▶) *T*_min_ = 0.973, *T*_max_ = 0.979
13086 measured reflections
2998 independent reflections
1941 reflections with *I* > 2σ(*I*)
*R*_int_ = 0.059

#### Refinement

*R*[*F* ^2^ > 2σ(*F* ^2^)] = 0.054
*wR*(*F* ^2^) = 0.148
*S* = 1.02
2998 reflections
190 parameters
H-atom parameters constrained
Δρ_max_ = 0.15 e Å^−3^
Δρ_min_ = −0.25 e Å^−3^

### 

Data collection: *CrystalClear* (Rigaku, 2005 ▶); cell refinement: *CrystalClear*; data reduction: *CrystalClear*; program(s) used to solve structure: *SHELXS97* (Sheldrick, 2008 ▶); program(s) used to refine structure: *SHELXL97* (Sheldrick, 2008 ▶); molecular graphics: *ORTEP-3 for Windows* (Farrugia, 1997 ▶) and *PLATON* (Spek, 2009 ▶); software used to prepare material for publication: *SHELXL97*.

## Supplementary Material

Crystal structure: contains datablocks I, global. DOI: 10.1107/S1600536809028438/hk2742sup1.cif

Structure factors: contains datablocks I. DOI: 10.1107/S1600536809028438/hk2742Isup2.hkl

Additional supplementary materials:  crystallographic information; 3D view; checkCIF report

## Figures and Tables

**Table 1:** Hydrogen-bond geometry (Å, °)

*D*—H⋯*A*	*D*—H	H⋯*A*	*D*⋯*A*	*D*—H⋯*A*
N2—H2*A*⋯O1	0.86	1.91	2.580 (2)	133
C12—H12*A*⋯O1^i^	0.93	2.45	3.362 (2)	166

Symmetry code: (i) 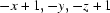.

**Fig. 1.**
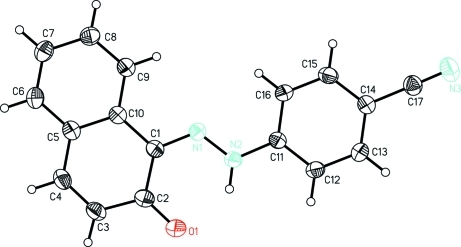
The molecular structure of the title molecule with the atom-numbering scheme. Hydrogen bond is shown as dashed line.

**Fig. 2.**
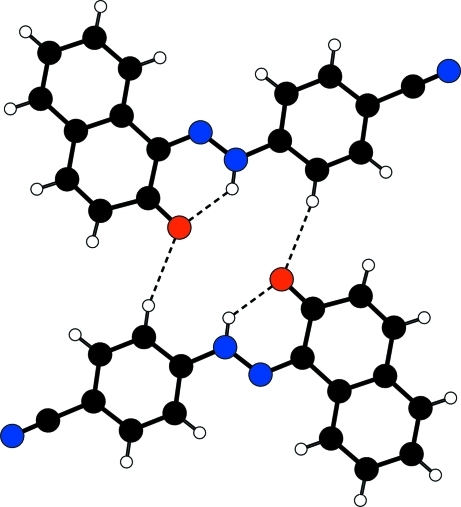
A partial packing diagram. Hydrogen bonds are shown as dashed lines.

## supplementary crystallographic information

### Comment

Azo compounds are characterized by the azo linkage (–N=N–) and are very important in the fields of dyes, pigments and advanced materials (Lee *et al.*, 2004; Oueslati *et al.*, 2004). We report herein the crystal structure of the title compound, obtained through the diazotization of 4-aminobenzonitrile followed by a coupling reaction with 2-naphthol.

In the molecule of the title compound (Fig. 1), the bond lengths (Allen *et al.*,  1987) and angles are within normal ranges. Rings A (C1-C5/C10), B (C5-C10) and C (C11-C16) are, of course, planar, and they are oriented at dihedral angles of A/B = 2.32 (3), A/C = 2.58 (3) and B/C = 4.59 (3) °. The naphthalene ring system is planar with a maximum deviation of 0.029 (3) Å for atom C5. Intramolecular N-H···O hydrogen bond (Table 1) results in the formation of planar six-membered ring D (O1/N1/N2/C1/C2/H2A), which is oriented with respect to rings A, B and C at dihedral angles of A/D = 1.12 (3), B/D = 3.29 (3) and C/D = 1.47 (3) °. So, rings A, B, C and D are almost coplanar.

In the crystal structure, intermolecular C-H···O interactions link the molecules into centrosymmetric dimers (Fig. 2), in which they may be effective in the stabilization of the structure.

### Experimental

The title compound was prepared according to a literature method (Rădulescu *et al.*, 2006). Crystals suitable for X-ray analysis were obtained by slow evaporation of an ethanol solution.

### Refinement

H atoms were positioned geometrically with N-H = 0.86 Å (for NH) and C-H = 0.93 Å for aromatic H atoms, respectively, and constrained to ride on their parent atoms, with U_iso_(H) = 1.2U_eq_(C,N).

### Crystal data

**Table:**

C_17_H_11_N_3_O	*F*(000) = 568
*M**_r_* = 273.29	*D*_x_ = 1.386 Mg m^−^^3^
Monoclinic, *P*2_1_/*c*	Mo *K*α radiation, λ = 0.71073 Å
Hall symbol: -P 2ybc	Cell parameters from 1658 reflections
*a* = 5.2673 (11) Å	θ = 3.2–28.9°
*b* = 9.910 (2) Å	µ = 0.09 mm^−^^1^
*c* = 25.239 (6) Å	*T* = 294 K
β = 96.13 (3)°	Block, red
*V* = 1309.9 (5) Å^3^	0.35 × 0.10 × 0.10 mm
*Z* = 4	

### Data collection

**Table:**

Rigaku SCXmini diffractometer	2998 independent reflections
Radiation source: fine-focus sealed tube	1941 reflections with *I* > 2σ(*I*)
graphite	*R*_int_ = 0.059
Detector resolution: 13.6612 pixels mm^-1^	θ_max_ = 27.5°, θ_min_ = 3.2°
ω scans	*h* = −6→6
Absorption correction: multi-scan (*CrystalClear*; Rigaku, 2005)	*k* = −12→12
*T*_min_ = 0.973, *T*_max_ = 0.979	*l* = −32→32
13086 measured reflections	

### Refinement

**Table:**

Refinement on *F*^2^	Primary atom site location: structure-invariant direct methods
Least-squares matrix: full	Secondary atom site location: difference Fourier map
*R*[*F*^2^ > 2σ(*F*^2^)] = 0.054	Hydrogen site location: inferred from neighbouring sites
*wR*(*F*^2^) = 0.148	H-atom parameters constrained
*S* = 1.02	*w* = 1/[σ^2^(*F*_o_^2^) + (0.0717*P*)^2^ + 0.0861*P*] where *P* = (*F*_o_^2^ + 2*F*_c_^2^)/3
2998 reflections	(Δ/σ)_max_ < 0.001
190 parameters	Δρ_max_ = 0.15 e Å^−^^3^
0 restraints	Δρ_min_ = −0.25 e Å^−^^3^

### Special details

**Table:**

Geometry. All e.s.d.'s (except the e.s.d. in the dihedral angle between two l.s. planes) are estimated using the full covariance matrix. The cell e.s.d.'s are taken into account individually in the estimation of e.s.d.'s in distances, angles and torsion angles; correlations between e.s.d.'s in cell parameters are only used when they are defined by crystal symmetry. An approximate (isotropic) treatment of cell e.s.d.'s is used for estimating e.s.d.'s involving l.s. planes.
Refinement. Refinement of *F*^2^ against ALL reflections. The weighted *R*-factor *wR* and goodness of fit *S* are based on *F*^2^, conventional *R*-factors *R* are based on *F*, with *F* set to zero for negative *F*^2^. The threshold expression of *F*^2^ > σ(*F*^2^) is used only for calculating *R*-factors(gt) *etc*. and is not relevant to the choice of reflections for refinement. *R*-factors based on *F*^2^ are statistically about twice as large as those based on *F*, and *R*- factors based on ALL data will be even larger.

### Fractional atomic coordinates and isotropic or equivalent isotropic displacement parameters (Å^2^)

**Table:**

	*x*	*y*	*z*	*U*_iso_*/*U*_eq_	
O1	0.2630 (3)	−0.03169 (14)	0.45197 (5)	0.0627 (4)	
N1	0.3499 (3)	0.15188 (14)	0.36889 (6)	0.0424 (4)	
N2	0.5123 (3)	0.16008 (14)	0.41170 (5)	0.0444 (4)	
H2A	0.4994	0.1072	0.4383	0.053*	
N3	1.4464 (4)	0.62761 (18)	0.42541 (8)	0.0731 (5)	
C1	0.1628 (3)	0.06230 (17)	0.36607 (7)	0.0427 (4)	
C2	0.1200 (4)	−0.03101 (18)	0.40895 (7)	0.0503 (5)	
C3	−0.0963 (4)	−0.12070 (19)	0.39970 (8)	0.0576 (5)	
H3A	−0.1284	−0.1821	0.4261	0.069*	
C4	−0.2519 (4)	−0.11777 (18)	0.35418 (8)	0.0542 (5)	
H4A	−0.3884	−0.1777	0.3501	0.065*	
C5	−0.2179 (3)	−0.02633 (17)	0.31131 (7)	0.0456 (4)	
C6	−0.3895 (3)	−0.02176 (19)	0.26515 (7)	0.0534 (5)	
H6A	−0.5266	−0.0814	0.2614	0.064*	
C7	−0.3597 (4)	0.0688 (2)	0.22535 (7)	0.0554 (5)	
H7A	−0.4760	0.0711	0.1949	0.066*	
C8	−0.1543 (4)	0.15722 (19)	0.23078 (7)	0.0514 (5)	
H8A	−0.1329	0.2185	0.2037	0.062*	
C9	0.0175 (3)	0.15515 (18)	0.27562 (7)	0.0474 (4)	
H9A	0.1544	0.2149	0.2786	0.057*	
C10	−0.0106 (3)	0.06409 (16)	0.31707 (6)	0.0410 (4)	
C11	0.7076 (3)	0.25635 (16)	0.41359 (6)	0.0398 (4)	
C12	0.8755 (3)	0.26618 (19)	0.45927 (7)	0.0505 (5)	
H12A	0.8579	0.2089	0.4878	0.061*	
C13	1.0691 (4)	0.36029 (19)	0.46282 (7)	0.0514 (5)	
H13A	1.1819	0.3666	0.4937	0.062*	
C14	1.0948 (3)	0.44545 (17)	0.42018 (7)	0.0446 (4)	
C15	0.9273 (3)	0.43388 (19)	0.37409 (7)	0.0509 (5)	
H15A	0.9456	0.4904	0.3453	0.061*	
C16	0.7350 (3)	0.33980 (18)	0.37050 (7)	0.0477 (4)	
H16A	0.6240	0.3321	0.3394	0.057*	
C17	1.2921 (4)	0.5465 (2)	0.42332 (7)	0.0533 (5)	

### Atomic displacement parameters (Å^2^)

**Table:**

	*U*^11^	*U*^22^	*U*^33^	*U*^12^	*U*^13^	*U*^23^
O1	0.0707 (9)	0.0608 (8)	0.0547 (8)	−0.0102 (7)	−0.0025 (7)	0.0170 (6)
N1	0.0445 (8)	0.0398 (8)	0.0427 (8)	0.0009 (6)	0.0035 (6)	−0.0017 (6)
N2	0.0509 (9)	0.0406 (8)	0.0417 (8)	−0.0023 (6)	0.0044 (7)	0.0056 (6)
N3	0.0691 (11)	0.0661 (12)	0.0849 (14)	−0.0198 (10)	0.0124 (10)	−0.0066 (10)
C1	0.0452 (10)	0.0373 (9)	0.0464 (9)	0.0016 (7)	0.0087 (8)	−0.0003 (7)
C2	0.0557 (11)	0.0443 (10)	0.0515 (11)	0.0010 (8)	0.0091 (9)	0.0054 (8)
C3	0.0612 (12)	0.0487 (11)	0.0638 (12)	−0.0088 (9)	0.0104 (10)	0.0127 (9)
C4	0.0503 (11)	0.0446 (10)	0.0680 (13)	−0.0085 (8)	0.0084 (10)	0.0016 (9)
C5	0.0459 (10)	0.0413 (10)	0.0505 (10)	−0.0009 (8)	0.0100 (8)	−0.0057 (8)
C6	0.0474 (10)	0.0532 (11)	0.0591 (12)	−0.0049 (9)	0.0031 (9)	−0.0092 (9)
C7	0.0531 (11)	0.0616 (12)	0.0501 (11)	0.0027 (10)	−0.0005 (9)	−0.0086 (9)
C8	0.0563 (11)	0.0534 (11)	0.0448 (10)	0.0023 (9)	0.0064 (9)	−0.0002 (8)
C9	0.0486 (10)	0.0461 (10)	0.0481 (10)	−0.0027 (8)	0.0084 (8)	−0.0017 (8)
C10	0.0426 (9)	0.0376 (9)	0.0436 (9)	0.0025 (7)	0.0087 (7)	−0.0034 (7)
C11	0.0425 (9)	0.0354 (9)	0.0421 (9)	0.0019 (7)	0.0080 (7)	−0.0002 (7)
C12	0.0621 (12)	0.0494 (11)	0.0392 (9)	−0.0036 (9)	0.0021 (9)	0.0042 (8)
C13	0.0551 (11)	0.0540 (11)	0.0436 (10)	−0.0057 (9)	−0.0016 (8)	−0.0025 (8)
C14	0.0435 (9)	0.0422 (9)	0.0488 (10)	0.0004 (8)	0.0090 (8)	−0.0023 (8)
C15	0.0520 (11)	0.0498 (10)	0.0517 (10)	−0.0005 (9)	0.0088 (9)	0.0122 (8)
C16	0.0483 (10)	0.0505 (10)	0.0429 (9)	−0.0007 (8)	−0.0007 (8)	0.0074 (8)
C17	0.0523 (11)	0.0541 (11)	0.0543 (11)	−0.0045 (9)	0.0098 (9)	−0.0034 (9)

### Geometric parameters (Å, °)

**Table:**

O1—C2	1.254 (2)	C7—C8	1.387 (3)
N1—N2	1.3072 (19)	C7—H7A	0.9300
N1—C1	1.323 (2)	C8—C9	1.372 (2)
N2—C11	1.400 (2)	C8—H8A	0.9300
N2—H2A	0.8600	C9—C10	1.401 (2)
N3—C17	1.141 (2)	C9—H9A	0.9300
C1—C10	1.457 (2)	C11—C12	1.380 (2)
C1—C2	1.459 (2)	C11—C16	1.386 (2)
C2—C3	1.444 (3)	C12—C13	1.378 (2)
C3—C4	1.338 (3)	C12—H12A	0.9300
C3—H3A	0.9300	C13—C14	1.386 (2)
C4—C5	1.437 (3)	C13—H13A	0.9300
C4—H4A	0.9300	C14—C15	1.387 (2)
C5—C6	1.397 (2)	C14—C17	1.439 (3)
C5—C10	1.408 (2)	C15—C16	1.373 (2)
C6—C7	1.369 (3)	C15—H15A	0.9300
C6—H6A	0.9300	C16—H16A	0.9300
			
N2—N1—C1	120.28 (14)	C7—C8—H8A	119.6
N1—N2—C11	118.95 (14)	C8—C9—C10	120.83 (17)
N1—N2—H2A	120.5	C8—C9—H9A	119.6
C11—N2—H2A	120.5	C10—C9—H9A	119.6
N1—C1—C10	115.69 (15)	C9—C10—C5	118.39 (16)
N1—C1—C2	123.97 (16)	C9—C10—C1	122.33 (15)
C10—C1—C2	120.31 (15)	C5—C10—C1	119.27 (15)
O1—C2—C3	121.83 (17)	C12—C11—C16	120.16 (16)
O1—C2—C1	121.30 (16)	C12—C11—N2	118.66 (15)
C3—C2—C1	116.86 (17)	C16—C11—N2	121.18 (16)
C4—C3—C2	121.70 (18)	C13—C12—C11	120.41 (16)
C4—C3—H3A	119.1	C13—C12—H12A	119.8
C2—C3—H3A	119.1	C11—C12—H12A	119.8
C3—C4—C5	123.09 (17)	C12—C13—C14	119.67 (17)
C3—C4—H4A	118.5	C12—C13—H13A	120.2
C5—C4—H4A	118.5	C14—C13—H13A	120.2
C6—C5—C10	119.42 (16)	C13—C14—C15	119.65 (16)
C6—C5—C4	121.78 (16)	C13—C14—C17	120.72 (17)
C10—C5—C4	118.76 (16)	C15—C14—C17	119.63 (16)
C7—C6—C5	121.22 (17)	C16—C15—C14	120.67 (16)
C7—C6—H6A	119.4	C16—C15—H15A	119.7
C5—C6—H6A	119.4	C14—C15—H15A	119.7
C6—C7—C8	119.42 (18)	C15—C16—C11	119.44 (16)
C6—C7—H7A	120.3	C15—C16—H16A	120.3
C8—C7—H7A	120.3	C11—C16—H16A	120.3
C9—C8—C7	120.72 (18)	N3—C17—C14	179.1 (2)
C9—C8—H8A	119.6		

### Hydrogen-bond geometry (Å, °)

**Table:**

*D*—H···*A*	*D*—H	H···*A*	*D*···*A*	*D*—H···*A*
N2—H2A···O1	0.86	1.91	2.580 (2)	133
C12—H12A···O1^i^	0.93	2.45	3.362 (2)	166

Symmetry codes: (i) −*x*+1, −*y*, −*z*+1.
